# German mammography screening program: adherence, characteristics of (non-)participants and utilization of non-screening mammography—a longitudinal analysis

**DOI:** 10.1186/s12889-023-16589-5

**Published:** 2023-08-31

**Authors:** Miriam Heinig, Wiebke Schäfer, Ingo Langner, Hajo Zeeb, Ulrike Haug

**Affiliations:** 1 Department of Clinical Epidemiology, Leibniz Institute for Prevention Research and Epidemiology – BIPS, Achterstraße 30, 28359 Bremen, Germany; 2 Department of Prevention and Evaluation, Leibniz Institute for Prevention Research and Epidemiology – BIPS, Bremen, Germany; 3 Faculty of Human and Health Sciences, University of Bremen, Bremen, Germany

**Keywords:** Breast cancer screening, Adherence, Longitudinal study, Mammography, Gray screening, Comorbidity, Claims data

## Abstract

**Background:**

In Germany, all women aged 50–69 have been invited to biennial mammography screening since 2009. We aimed to assess longitudinal adherence over ten years in women aged 50 in 2009 and characterize the different adherence groups.

**Methods:**

Using the German Pharmacoepidemiological Research Database (GePaRD, ~ 20% of the German population), we included women aged 50 in 2009 (baseline) with continuous health insurance coverage and without breast cancer or in-situ-carcinoma. We followed them until age 59 and categorized them according to mammography screening participation into the following groups: never, 1–2, 3–4, 5–6 times. We characterized these groups, inter alia, regarding the use of other preventive measures, non-screening mammography (i.e., mammography outside the organized screening program) and menopausal hormone therapy.

**Results:**

Overall, 82,666 women were included. Of these, 27.6% never participated in the screening program, 15.1% participated 1–2 times, 31.7% participated 3–4 times and 25.6% participated regularly (5–6 times). Among regular participants, 91% utilized other preventive measures (e.g., cervical cancer screening, general health checkup) before baseline as compared to 66% among non-participants. Menopausal hormone therapy was least common among non-participants (11% vs. 18% among regular participants). Among non-participants, the proportions using ≥ 1, ≥ 2, and ≥ 3 non-screening mammographies between age 50–59 were 25%, 18%, and 15%, respectively.

**Conclusions:**

Using a large cohort based on claims data, this study provides novel insights into longitudinal adherence to the mammography screening program and the use of mammography outside of the program in Germany. Between age 50–59, 57% of eligible women participated at least three times in the German mammography screening program and 28% (~ 3 in 10 women) never participated. Among non-participants, 15% had at least three non-screening mammographies during this period, indicating potential gray screening. Participants more often utilized other preventive measures as compared to non-participants.

**Supplementary Information:**

The online version contains supplementary material available at 10.1186/s12889-023-16589-5.

## Introduction

Mammography screening aims at detecting breast cancer before it presents with symptoms in order to improve prognosis through early treatment and thus prevent death from the disease. Eleven randomized controlled trials (RCTs) evaluating the effectiveness of mammography screening starting in the early 1960’s found a mortality reduction from breast cancer of about 20% [1]. In most of these trials, overall participation among women randomized to the intervention arm was high at about 80% [2, 3], i.e., the intention-to-treat effect observed in these trials is based on these adherence rates. Given the convincing RCT evidence, organized mammography screening programs have been established in European and many other countries around the world [4]. Core aspects of organized screening programs include regular invitations, quality assurance as well as program monitoring and evaluation. When evaluating these programs, adherence is an important aspect. There is a large number of cross-sectional studies reporting on the proportion of women with vs. without a screening mammography in the previous two years (i.e., the regular screening interval), or on anytime participation [5–8]. However, longitudinal studies investigating patterns of adherence over time are less commonly performed [9–11]. Knowledge is also limited as to whether irregular and regular participants (as determined by longitudinal analyses) are similar with respect to factors relevant for self-selection to screening [12], and which proportion of never participants undergo mammography outside the program.

There is also a lack of studies on these aspects in Germany, where a mammography screening program was implemented in 2005 and reached full population coverage in 2009 [13]. Women aged 50–69 are invited biennially to undergo a mammography. Within the program, screening adherence is assessed cross-sectionally for each screening round [14], and only basic demographic data are available to characterize participants and non-participants. Health claims data may fill this gap as they contain information on screening mammography, other healthcare utilization including non-screening mammography as well as on comorbidity.

We therefore aimed to conduct longitudinal analyses on ten-year adherence to mammography screening in Germany in an exemplary cohort of women aged 50 in 2009 and characterize regular and irregular participants as well as non-participants based on health claims data.

## Methods

### Data source

We used the German Pharmacoepidemiological Research Database (GePaRD) which is based on claims data from four statutory health insurance providers in Germany. GePaRD currently includes information on approximately 25 million persons who have been insured with one of the participating providers since 2004 or later. In addition to demographic data, GePaRD contains information on drug dispensations as well as outpatient (i.e., from general practitioners and specialists) and inpatient services and diagnoses. Per data year, there is information on approximately 20% of the general population and all geographical regions of Germany are represented [15]. The coverage varies between geographical regions: It tends to be higher in the northern and western part than in the southern and eastern part of Germany. Core characteristics of the German health insurance system are uniform access to all levels of care and free choice of providers.

### Study design

In the present study, we considered the birth cohort of women turning 50 years in 2009, i.e., women born in 1959 for inclusion (*n* = 123,195). We focused on this exemplary cohort because in Germany, mammography screening starts at age 50 and the program was fully implemented in 2009 [13], so this birth cohort was the first with full access to the program. We excluded women who were not continuously insured in the two years prior to 2009 (i.e., 2007–2008). This pre-observation period was required to characterize the women before they became eligible for the program. Further, we excluded women who could not be continuously observed from 2009 to 2018 as we aimed to assess longitudinal adherence patterns. We also excluded women who had any in- or outpatient diagnosis code for invasive breast cancer (ICD-10-GM “C50”) or carcinoma-in-situ of the breast (ICD-10-GM “D05”) between 2007 and 2018 to focus the analysis on women who were eligible to participate in mammography screening throughout the whole ten-year period from 2009–2018. Lastly, we excluded women who had an (erroneous) coding of screening mammography before the eligible age (i.e., before age 50). Applying these exclusion criteria to the initial cohort led to a final sample cohort size of 82,666 women (Additional file 1, Fig. 1A).

### Assessment of screening adherence and other characteristics

Participation in the mammography screening program was assessed by the respective outpatient claims code. We classified the women into the following categories according to how often they participated in screening mammography from the year they turned 50 to the year they turned 59 (i.e., 2009–2018): Never, 1–2 times, 3–4 times or 5–6 times. We defined the upper category as “5–6 times” based on the consideration that the biennial schedule leads to five screening offers between age 50–59, but there were also some women participating six times during this period.

The level of education as an indicator of the socioeconomic status was estimated based on a previously developed algorithm [16]. This algorithm uses information on the highest school degree or the occupation to categorize persons into different educational levels. For the present analyses, we used the categories “basic secondary degree/secondary degree or missing/unknown information” and “higher education”. As discussed by Asendorf et al. the information on education is not missing at random but women aged 50 years or older with missing information on education most likely tend to have lower education [16], so we included them in this category. Regarding utilization of other preventive measures (cervical cancer screening, skin cancer screening, health checkup, influenza vaccination) we assessed whether the respective code was recorded at least once during the pre-observation period (i.e., 2007–2008) (yes/no).

The proportion of women utilizing non-screening mammography (i.e., mammographies outside the organized screening program) was assessed based on the respective codes, which are different from the codes for screening mammography.

The prevalence of comorbidities (any severe comorbidity, treatment for hypertension, obesity, treatment with antidepressants/antipsychotics) was assessed during the pre-observation period (i.e., 2007–2008) based on previously developed algorithms ensuring a high specificity of disease definition. In addition, we assessed whether there was at least one code for alcohol abuse, tobacco abuse or prescription of menopausal hormone therapy, which are risk factors for breast cancer [17], or a diagnosis of glaucoma at least once during the pre-observation period (yes/no). Glaucoma was of interest because it has been used as an indicator of higher healthcare utilization in other studies [18]. We also assessed whether there was a code for a family history of breast cancer any time during the women’s database history (yes/no).

### Data analysis

We first categorized the women according to their participation in mammography screening (see above). We then conducted descriptive analyses stratified by these categories for the characteristics described above. Regarding the use of non-screening mammographies, two different analyses were conducted: First, we described the use of non-screening mammography in the two years before age 50/55/59 to determine how the use of these examinations changed after women became eligible for the organized screening program. Second, we described the use of non-screening mammographies for the whole period from age 50–59 in terms of total number of examinations and intervals.

We calculated 95% confidence intervals for all proportions and means. In sensitivity analyses, we varied the assessment period for characteristics that were—in the main analysis—assessed during the two-year pre-observation period because characteristics may change over time. Instead of age 48/49 (2007/2008) in the main analysis, the alternative assessment periods were age 53/54 (2012/2013) and age 57/58 (2016/2017) in the sensitivity analyses. In another sensitivity analysis, we characterized women who participated in mammography screening only once during the ten-year period to assess whether their characteristics differed from the other adherence groups.

All analyses were performed using SAS® software (SAS Institute, version 9.4, NC, USA).

## Results

Out of 123,195 women born in 1959, 82,666 women were included in the analysis. The selection of the study population is illustrated in Additional file 1 (Figure A1). The criterion “not continuously insured from 2007–2018” led to most exclusions (*n* = 35,286). Women excluded due to this criterion are characterized in Additional file 1 (Tables A1-A2). While there were no substantial differences in comorbidity and other characteristics, the proportion with higher education was lower among excluded as compared to included women (26% vs. 37%).

Between age 50–59, 27.6% of included women never participated, 15.1% participated 1–2 times, 31.7% participated 3–4 times, and 25.6% participated regularly (5–6 times). Table 1 shows the distribution of age at first mammography screening and educational level among included women stratified by screening adherence. Women participating 1–2 times were on average two years older at their first screen than women participating 5–6 times (53 years vs. 51 years). The educational level did not differ by screening adherence: The proportion of women with higher education was 36%–39% across all groups.

**Table 1:** Distribution of included women regarding ten-year adherence to mammography screening and characterization of adherence groups by age and educational level^a^

	**Number of participations in mammography screening**											
	**Never**			**1–2 times**			**3–4 times**			**5–6 times**		
	**N**	**%**	**95% CI**	**N**	**%**	**95% CI**	**N**	**%**	**95% CI**	**N**	**%**	**95% CI**
	**22,786**	**(27.6%)**	**(27.3, 27.9)**	**12,521**	**(15.1%)**	**(14.9, 15.4)**	**26,166**	**(31.7%)**	**(31.3, 32.0)**	**21,193**	**(25.6%)**	**(25.3, 25.9)**
**Age at first screen**												
Mean (SD)	N/A	N/A	N/A	53.0	(2.5)	(52.9, 53.0)	51.5	(1.2)	(51.5, 51.6)	50.5	(0.5)	(50.5, 50.5)
Median (IQR)	N/A	N/A	-	52.0	(51–55)	-	51.0	(51–52)	-	50.0	(50–51)	-
**Education**												
Basic secondary degree/secondary degree or missing information	14,647	(64.3%)	(63.7, 64.9)	7,697	(61.5%)	(60.6, 62.3)	16,798	(64.2%)	(63.6, 64.8)	13,373	(63.1%)	(62.4, 63.7)
Higher education	8,139	(35.7%)	(35.1, 36.3)	4,824	(38.5%)	(37.7, 39.4)	9,368	(35.8%)	(35.2, 36.4)	7,820	(36.9%)	(36.3, 37.6)

*CI* Confidence interval, *SD* Standard deviation, *IQR* Interquartile range, *N/A* Not applicable

^a^Non-stratified results for all included women are available in Additional file 1 (Table A8)

Table 2 shows the use of other preventive measures as well as the presence of comorbidities and other factors at age 50 stratified by screening adherence. For all preventive measures, there was a gradient in the uptake correlated with whether or not and how regularly women participated in mammography screening. For example, cervical cancer screening was used by 85% of regular participants and by 54% of non-participants. The proportion of women undergoing a health checkup, typically conducted by general physicians, was 33% among non-participants and 48% among regular participants. Influenza vaccination was also less frequently used among non-participants compared to regular participants (12% vs. 21%). Overall, 91% of regular participants had used at least one other type of preventive measure in the previous two years, compared to 66% of non-participants.

**Table 2:** Utilization of other preventive measures and prevalence of comorbidities and other characteristics, stratified by ten-year adherence to mammography screening^a^

	**Number of participations in mammography screening**											
	**Never**			**1–2 times**			**3–4 times**			**5–6 times**		
	**N**	**%**	**95% CI**	**N**	**%**	**95% CI**	**N**	**%**	**95% CI**	**N**	**%**	**95% CI**
	**22,786**	**(27.6%)**	**(27.3, 27.9)**	**12,521**	**(15.1%)**	**(14.9, 15.4)**	**26,166**	**(31.7%)**	**(31.3, 32.0)**	**21,193**	**(25.6%)**	**(25.3, 25.9)**
**Other screening and preventive measures**^**b**^												
Cervical cancer screening (PAP test)^c^	12,276	(53.9%)	(53.7, 54.5)	8,946	(71.4%)	(70.7, 72.7)	20,978	(80.2%)	(79.7, 80.7)	18,053	(85.2%)	(84.7, 85.7)
Skin cancer screening^d^	1,159	(5.1%)	(4.8, 5.4)	840	(6.7%)	(6.3, 7.2)	2040	(7.8%)	(7.5, 8.1)	1,899	(9.0%)	(8.6, 9.4)
Health checkup	7,414	(32.5%)	(31.9, 33.1)	5,056	(40.4%)	(39.5, 41.2)	11,450	(43.8%)	(43.2, 44.4)	10,230	(48.3%)	(47.6, 48.9)
Influenza vaccination	2,709	(11.9%)	(11.5, 12.3)	1,819	(14.5%)	(13.9, 15.2)	4,750	(18.2%)	(17.7, 18.6)	4,429	(20.9%)	(20.4, 21.5)
Any of these preventive measures	15,072	(66.1%)	(65.5, 66.8)	10,226	(81.7%)	(81.0, 82.3)	22,919	(87.6%)	(87.2, 88.0)	19,311	(91.1%)	(90.7, 91.5)
**Comorbidities**												
Any severe comorbidity^e^	1,590	(7.0%)	(6.7, 7.3)	970	(7.7%)	(7.3, 8.2)	1,875	(7.2%)	(6.9, 7.5)	1,507	(7.1%)	(6.8, 7.5)
Treatment for hypertension	2,641	(11.6%)	(11.2, 12.0)	1,570	(12.5%)	(12.0, 13.1)	3,767	(14.4%)	(14.0, 14.8)	3,209	(15.1%)	(14.7, 15.6)
Obesity	2,283	(10.0%)	(9.6, 10.4)	1,468	(11.7%)	(11.2, 12.3)	3,365	(12.9%)	(12.5, 13.3)	2,807	(13.2%)	(12.8, 13.7)
Glaucoma	816	(3.6%)	(3.3, 3.8)	512	(4.1%)	(3.8, 4.5)	1,340	(5.1%)	(4.9, 5.4)	1,260	(5.9%)	(5.6, 6.3)
Treatment with antidepressants	870	(3.8%)	(3.6, 4.1)	598	(4.8%)	(4.4, 5.2)	1,105	(4.2%)	(4.0, 4.5)	745	(3.5%)	(3.3, 3.8)
Treatment with antipsychotics	279	(1.2%)	(1.1, 1.4)	142	(1.1%)	(1.0, 1.3)	224	(0.9%)	(0.8, 1.0)	118	(0.6%)	(0.5, 0.7)
**Other characteristics**												
Alcohol abuse	433	(1.9%)	(1.7, 2.1)	271	(2.2%)	(1.9, 2.4)	323	(1.2%)	(1.1, 1.4)	174	(0.8%)	(0.7, 1.0)
Tobacco abuse	1,303	(5.7%)	(5.4, 6.0)	771	(6.2%)	(5.7, 6.6)	1,593	(6.1%)	(5.8, 6.4)	1,127	(5.3%)	(5.0, 5.6)
Menopausal hormone therapy	2,605	(11.4%)	(11.0, 11.9)	1,925	(15.4%)	(14.8, 16.0)	4,547	(17.4%)	(16.9, 17.8)	3,825	(18.4%)	(17.5, 18.6)
Family history of breast cancer	926	(4.1%)	(3.8, 4.3)	434	(3.5%)	(3.2, 3.8)	885	(3.4%)	(3.2, 3.6)	628	(3.0%)	(2.7, 3.2)

*CI* Confidence interval, *PAP* Papanicolaou test

^a^Non-stratified results for all included women are available in Additional file 1 (Table A9)

^b^Screening for colorectal cancer with the fecal occult blood test is offered from age 50 (age 55: additional offer of screening colonoscopy) and therefore not included here

^c^As part of screening for female cancers, cervical cancer screening can be combined with physical breast examination

^d^Codes for skin cancer screening were available starting in July 2008 only

^e^Any of the following: Liver disease, coronary heart disease, congestive heart failure, myocardial infarction, stroke, COPD, Hepatitis (B, C), renal insufficiency terminal), diabetes with organ damage, treated HIV, dementia, plegia

The proportion of women with any severe comorbidity was 7%–8% in all groups. Regular participants were more frequently treated for hypertension compared to non-participants (15% vs. 12%) and more frequently had a diagnosis of glaucoma (6% vs. 4%) and obesity (13% vs. 10%). The proportion of women who received treatment with antidepressants ranged between 4 and 5% across groups. A code indicating alcohol abuse was present in 1%–2% of women across all groups, and a code indicating heavy smoking was present in 5%–6% of women across all groups. Other comorbidities and those with very low prevalence are shown in Additional file 1 (Table A3). There was a gradient in the prevalence of menopausal hormone therapy use: it was highest among regular participants (18%) and lowest among non-participants (11%). A family history of breast cancer was rarely coded, both among non-participants (4%) and among regular participants (3%).

**Fig. 1:**
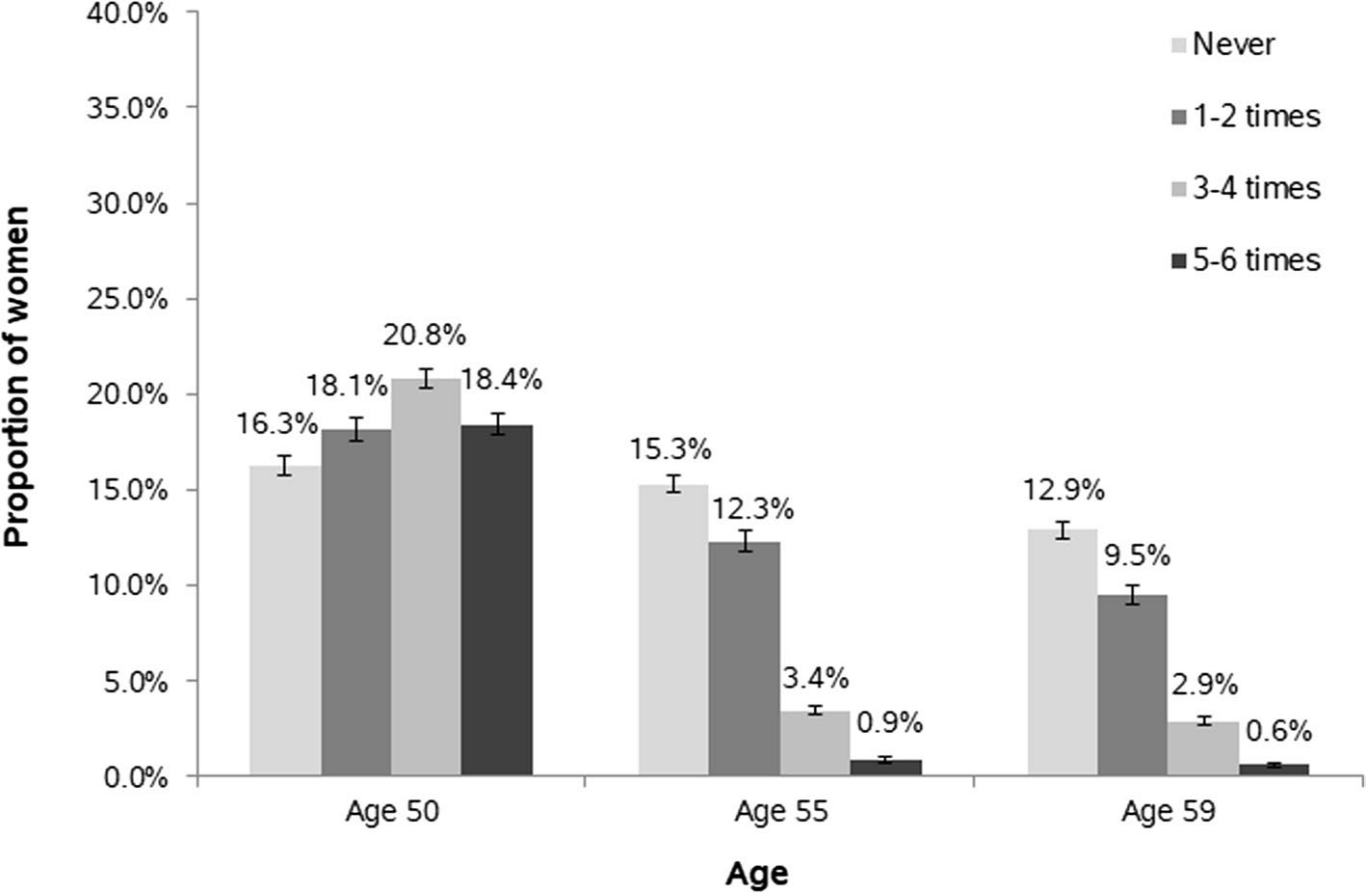
Proportion of women with a non-screening mammography in the two years prior to age 50/55/59 with 95% confidence interval, stratified by frequency of (longitudinal) adherence to mammography screening (never, 1–2 times, 3–4 times, 5–6 times from age 50–59)

Figure 1 shows the proportion of women with at least one non-screening mammography in the two years prior to ages 50/55/59, stratified by screening adherence. At age 50, the proportions ranged between 16 and 21% across all groups. At ages 55 and 59, this proportion decreased to ≤ 3% among women participating 3–4 or 5–6 times, respectively, while it was 10%–12% among those participating 1–2 times and 13%–15% among non-participants.

Among all non-participants, the proportions using ≥ 1, ≥ 2, and ≥ 3 non-screening mammographies between age 50–59 were 25%, 18%, and 15%, respectively (Table 3). Among non-participants who had at least one non-screening mammography between age 50–59, 47% had more than three non-screening mammographies in total from 2009 to 2018. Among regular participants, this proportion was 1%. The mean interval in years between non-screening mammographies was 1.9 years (± 1.0) among non-participants. Of the whole cohort, 21% did not have any non-screening or screening mammography between age 50–59.

**Table 3:** Total number of non-screening mammographies in all women, and characterization of the use of non-screening mammographies in women with at least one non-screening mammography between age 50–59, stratified by ten-year adherence to mammography screening^a^

	**Number of participations in mammography screening**											
	**Never**			**1–2 times**			**3–4 times**			**5–6 times**		
	**N**	**%**	**95% CI**	**N**	**%**	**95% CI**	**N**	**%**	**95% CI**	**N**	**%**	**95% CI**
**Total number of non-screening mammographies between age 50–59**												
	22,786			12,521			26,166			21,193		
≥ 1 mammography	5,668	(24.9%)	(24.3, 25.4)	3,480	(27.8%)	(27.0, 28.6)	5,704	(21.8%)	(21.3, 22.3)	978	(4.6%)	(4.3, 4.9)
≥ 2 mammographies	4,126	(18.1%)	(17.6, 18.6)	1,983	(15.8%)	(15.2, 16.5)	1,405	(5.4%)	(5.1, 5.6)	113	(0.5%)	(0.4, 0.6)
≥ 3 mammographies	3,333	(14.6%)	(14.2, 15.1)	1,258	(10.0%)	(9.5, 10.6)	369	(1.4%)	(1.3, 1.6)	30	(0.1%)	(0.1, 0.2)
**Among those with ≥ 1 non-screening mammography**												
	5,668			3,480			5,704			978		
Distribution of the total number of non-screening mammographies												
Mean (SD)	3.5	(2.3)	(3.5, 3.6)	2.3	(1.6)	(2.2, 3.4)	1.3	(0.7)	(1.3, 1.4)	1.2	(0.5)	(1.1, 1.2)
Median (IQR)	3	(1–5)	-	2	(1–3)	-	1	(1–1)	-	1	(1–1)	-
1 mammography	1,542	(27.2%)	(26.1, 28.4)	1,497	(43.0%)	(41.4, 44.7)	4,299	(75.4%)	(74.2, 76.5)	865	(88.4%)	(86.3, 90.3)
2–3 mammographies	1,477	(26.1%)	(24.9, 27.2)	1,304	(37.5%)	(35.9, 39.1)	1,288	(22.6%)	(21.5, 23.7)	104	(10.6%)	(8.9, 12.7)
> 3 mammographies	2,649	(46.7%)	(45.4, 48.0)	679	(19.5%)	(18.2, 20.9)	117	(2.1%)	(1.7, 2.5)	9	(0.9%)	(0.5, 1.7)
**Among those with ≥ 2 non-screening mammographies**												
	4,126			1,983			1,405			113		
Time interval between non-screening mammographies												
Mean (SD), years	1.9	(1.0)	(1.9, 1.9)	2.2	(1.3)	(2.1, 2.2)	2.4	(1.9)	(2.3, 2.5)	2.5	(1.7)	(2.2, 2.8)
Median (IQR), years	1.9	(1.1–2.3)	-	2.0	(1.2–2.5)	-	2.0	(1.1–2.8)	-	2.1	(1.8–2.5)	-

*CI* Confidence interval, *SD* Standard deviation, *IQR* Interquartile range

^a^Non-stratified results for all included women are available in Additional file 1 (Table A10)

As shown in Additional file 1 (Tables A4-A5), patterns regarding the prevalence of comorbidities or other factors and the use of other preventive measures across subgroups did not change when we assessed these covariates at ages 55 and 59 rather than at age 50. Another sensitivity analysis focusing on women participating only once (about 8% of all included women) showed that there were no relevant differences compared to the group of women participating once or twice (Additional file 1, Table A6).

## Discussion

This study is the first to give detailed insight into longitudinal adherence to mammography screening in Germany over ten years, including a characterization of the different adherence groups. Among 82,666 included women, 28% never participated in mammography screening between age 50–59, 15% participated 1–2 times, 32% participated 3–4 times, and 26% participated regularly (5–6 times). Among regular participants, 91% had used any other preventive measure (e.g., cervical cancer screening) before participation in mammography screening, while this proportion was only 66% among non-participants, i.e., adherence to mammography screening correlated with the use of other preventive healthcare offers. One quarter of non-participants had at least one non-screening mammography between age 50–59. The prevalence of severe comorbidities was similar in women participating vs. those not participating in mammography screening.

So far, information on longitudinal adherence to mammography screening has mainly been provided by studies conducted in Nordic countries. Out of 92,000 women invited to five rounds of mammography screening between 2008 and 2017 in the Capital Region of Denmark, 65% participated in all five rounds [9]. In a study from Sweden reporting on the first five rounds of the Stockholm mammography screening program introduced in 1989, 53% of women participated in all five rounds [10]. In a study from Norway among 48,000 women invited to ten rounds of mammography screening between 1996 and 2019, 52% participated in all ten rounds [11]. In all three studies, < 10% of included women never participated in the respective screening program [9–11]. In our study, the proportion of non-participants was higher (26%), while 47% participated regularly if we also considered women participating in four rounds (*n* = 17,345, 21%) in addition to those participating 5–6 times. This proportion is rather similar to the proportion of regular participants reported in the studies from Norway and Sweden. Defining only women who participated 5–6 times between age 50–59 as “regular participants” may indeed underestimate the proportion of regular participants. Although eligible women are expected to receive an invitation every two years, we could not assess whether there were delays leading to a reduced number of screening offers during the ten-year observation period. Furthermore, women starting screening not at age 50 but later and then participating regularly could not fulfill the criterion for “regular” participation (5–6 times) in our study, although this is correct in a strict sense as regular participation also means a timely start. These are further arguments why we think that the proportion of regular participants in Germany does not substantially differ from other countries.

It is usually assumed that persons participating in cancer screening are healthier or more health conscious than non-participants, also indicated by the term “healthy screenee bias”. Little is known as to whether or to which extent this assumption holds true for mammography screening in Germany. Available studies on this topic from Germany mostly focused on potential differences between participants and non-participants according to socioeconomic status [19–21]. In two large German health surveys, participation in mammography screening was higher in the middle education group as compared to the low and high education group, but these differences were not statistically significant [19, 20]. In our study, we did not observe any substantial difference in longitudinal screening adherence by educational level. However, among participants, we observed a markedly higher uptake of other preventive examinations conducted by gynecologists (cervical cancer screening, partly in combination with physical breast examination), suggesting that women who regularly visit a gynecologist are more sensitized to prevention of gynecological cancers. The utilization of other preventive measures (e.g., health check-ups, influenza vaccination) was also more common among women participating in mammography screening than among non-participants, suggesting a more pronounced preventive behavior among participants in general. Nonetheless, it should be noted that use of preventive measures among non-participants was still at a relatively high level.

We did not observe a difference in the prevalence of severe comorbidities between participants and non-participants, but—in line with previous reports [22]—conditions such as hypertension, glaucoma, and obesity were more common among regular participants compared to non- or irregular participants. This might indicate a more frequent interaction with the healthcare system leading to more frequent diagnosis and/or treatment rather than a true difference in the prevalence of these conditions. Regarding psychological comorbidities, a meta-analysis including 24 studies found lower attendance in mammography screening among women diagnosed with mental illness (combined odds ratio [OR] 0.72, 95% confidence interval 0.66–0.77) [23]. In our study, there was no clear association between screening adherence and treatment with antidepressants. We did, however, observe a pattern toward less frequent participation in women treated with antipsychotics, who in the meta-analysis by Mitchell et al. also showed much lower odds of participation (OR 0.55) than women with mood disorders (OR 0.83) [23]. Use of menopausal hormon therapy was more common among participants of mammography screening compared to non-participants, which is in line with findings from our recent systematic review comprising 32 studies from nine countries on the topic [24]. One reason might be the awareness of higher breast cancer risk among users of menopausal hormone therapy [25].

In the context of evaluating organized screening programs, it is important to know whether and to which extent screening also takes place outside the program (so-called gray screening). We therefore conducted detailed analyses on the utilization of non-screening mammography, which was possible given that mammographies conducted outside the program cannot be coded as screening mammographies. Some of these non-screening mammographies likely had a medical indication such as (other) benign breast diseases or follow-up diagnostics after a suspicious screen (information on indication is not available in our data). However, there were 15% of non-participants who had three or more non-screening mammographies from age 50–59, raising the suspicion that this was at least partly gray screening. Even though gray screening cannot be considered equal to program mammographies in terms of quality assurance, our finding still implies that the proportion of women who categorically refuse mammography screening is lower than assumed based on program data. Also, based on a large German health survey conducted in 2014/2015, Starker et al. reported that 19% of women who had a mammography in the past two years had this examination for reasons other than being invited to the screening program [19]. The fact that a family history of breast cancer was coded more often among non-participants than among participants might also be explained by the use of examinations outside the screening program. For women with a high genetic risk of breast cancer, there are special programs. Even though this does not apply to women with a simple family history of breast cancer, some physicians might propose examinations outside the program to these women.

The screening participation per round in Germany as reported based on cross-sectional data from the screening program is about 50% [14], In view of this relatively low proportion, it has often been argued that efforts are needed to increase participation in mammography screening. Our study shows that the proportion of women not at all participating in mammography screening is substantially lower than it seems based on the cross-sectional data. This could also imply that there is not much potential in campaigns raising awareness of mammography screening given that those who never participated between age 50–59 may have actively decided against screening. Interestingly, in a representative survey among 2,012 German statutory health insurance members (1,086 women) conducted in 2018, 21% of women stated a categorical refusal to participate in mammography screening [26]. This is rather similar to the proportion of women who never participated in mammography screening and did not undergo regular mammography outside the screening between age 50–59 as observed in our study. Also the lack of a socioeconomic gradient in mammography screening participation observed in our study (assessed based on education) may have implications. If this is confirmed by further studies that have more detailed information on the socioeconomic status including potential language barriers, then there may be little to gain from additional measures specifically addressing those with lower socioeconomic status. However, monitoring of participation according to socioeconomic status seems reasonable in order to be able to assess and respond to potential trends.

Some limitations should be kept in mind when interpreting the results of this study. First, life style factors are hardly captured in claims data. We considered codes for alcohol and tobacco abuse, but these are extreme forms of alcohol drinking and smoking which are only coded if the conditions (or associated diseases) prompt interaction with the healthcare system. The same may hold true for obesity but a recent study assessing the prevalence of obesity based on both claims and survey data did not suggest a pronounced underestimation when using claims data [16]. Second, among women excluded due to a lack of continuous health insurance coverage, the proportion with higher education was ten percentage points lower as compared to included women. However, because we did not observe a difference in screening adherence by educational level, we do not expect that this has biased results on longitudinal sceening adherence (this is further supported by a supplemental analysis described in Additional file 1, Table A7.) Third, to focus on eligible women, we excluded women diagnosed with invasive or in-situ breast cancer. An alternative would have been to do a person-time analysis and consider them until diagnosis but we do not think that this would have changed our results because the proportion of women excluded due to this criterion was small. Furthermore, prior analyses among breast cancer patients in Germany did not provide any indicators that they are special regarding their screening behavior [27]. Fourth, although all geographical regions in Germany are represented in our database, it does not have full population coverage and there is some variation in the coverage between regions. However, the regions with a higher coverage in our database include both federal states with a higher uptake (e.g., Mecklenburg-Western Pomerania) and a lower uptake (e.g., Schleswig–Holstein) of mammography screening according to program data [14]. In addition, when we analyzed our data in a way that facilitates comparison with the program data for the whole of Germany (i.e., participation per round) the average proportion of women participating in the program was 53% (data not shown) and thus rather similar to the proportion of about 50% reported by the program [14, 28].

To the best of our knowledge, this is the first study to report comprehensively on longitudinal participation in the German mammography screening program as well as on characteristics of participants and non-participants. Due to the nature of claims data, our study is free of non-responder and recall bias. Further, as claims data contain—unlike the program data—also information on non-screening mammographies, we were able to conduct detailed analyses in this regard and estimate the potential role of gray screening. Once further follow-up data are available, it will be interesting to examine longitudinal screening adherence beyond the age period 50 to 59 years and in other calendar years.

In conclusion, using a large cohort based on claims data, this study provides novel insights into longitudinal adherence to the mammography screening program and the use of mammography outside of the program in Germany. Between age 50–59, 57% of eligible women participated at least three times in the German mammography screening program and 28% (~ 3 in 10 women) never participated. Among non-participants, 15% had at least three non-screening mammographies during this period, indicating potential gray screening. Participants more often utilized other preventive measures as compared to non-participants.

### Supplementary Information

**Additional file 1: Figure A1.** Flowchart of selection of the study population. **Table A1.** Distribution of women with birth year 1959 and at least one day of insurance in 2009 excluded due to lack of continuous insurance from 2007-2018 according to educational level. **Table A2.** Utilization of other preventive measures and prevalence of comorbidities and other characteristics among women excluded due to lack of continuous insurance from 2007-2018 with information on covariates at age 48 and/or 49. **Table A3.** Prevalence of comorbidities and other characteristics, stratified by ten-year adherence to mammography screening. **Tables A4-A5.** Utilization of other preventive measures and prevalence of comorbidities and other characteristics at age 55 and 59, stratified by ten-year adherence to mammography screening. **Table A6.** Utilization of other preventive measures and prevalence of comorbidities and other characteristics, stratified by ten-year adherence to mammography screening (one-time only vs. 1–2 times). **Table A7.** Supplemental analysis regarding educational level and adherence to mammography screening. **Table A8.** Characterization of all included women by age and educational level. **Table A9.** Utilization of other preventive measures and prevalence of comorbidities and other characteristics in all included women. **Table A10.** Total number of non-screening mammographies, and characterization of the use of non-screening mammographies in women with at least one non-screening mammography between age 50–59, in all included women.

## Data Availability

As we are not the owners of the data we are not legally entitled to grant access to the data of the German Pharmacoepidemiological Research Database (GePaRD). In accordance with German data protection regulations, access to the data is granted only to employees of the Leibniz Institute for Prevention Research and Epidemiology – BIPS on the BIPS premises and in the context of approved research projects. The data that support the findings of this study are available from BIPS, but restrictions apply to the availability of these data, which were used under license for the current study, and so are not publicly available. Data are however available from the authors upon reasonable request and with permission of BIPS. The data protection concept of GePaRD must be followed. Please contact the corresponding author (heinig@leibniz-bips.de) for inquiries related to the data used in this study.

## Abbreviations

GePaRD: German Pharmacoepidemiological Research Database
ICD-10-GM: International Classification of Diseases 10. Revision – German Modification
PAP: papanicolaou test
CI: Confidence interval
