# Supplementary material for: German mammography screening program: adherence, characteristics of (non-)participants and utilization of non-screening mammography—a longitudinal analysis
Source: BMC Public Health. 2023 Aug 31;23:1678. doi: 10.1186/s12889-023-16589-5 (PMC10469853; doi:10.1186/s12889-023-16589-5)
Supplement: Supplementary file 1 — Additional file 1: Figure A1. Flowchart of selection of the study population. Table A1. Distribution of women with birth year 1959 and at least one day of insurance in 2009 excluded due to lack of continuous insurance from 2007-2018 according to educational level. Table A2. Utilization of other preventive measures and prevalence of comorbidities and other characteristics among women excluded due to lack of continuous insurance from 2007-2018 with information on covariates at age 48 and/or 49. Table A3. Prevalence of comorbidities and other characteristics, stratified by ten-year adherence to mammography screening. Tables A4-A5. Utilization of other preventive measures and prevalence of comorbidities and other characteristics at age 55 and 59, stratified by ten-year adherence to mammography screening. Table A6. Utilization of other preventive measures and prevalence of comorbidities and other characteristics, stratified by ten-year adherence to mammography screening (one-time only vs. 1–2 times). Table A7. Supplemental analysis regarding educational level and adherence to mammography screening. Table A8. Characterization of all included women by age and educational level. Table A9. Utilization of other preventive measures and prevalence of comorbidities and other characteristics in all included women. Table A10. Total number of non-screening mammographies, and characterization of the use of non-screening mammographies in women with at least one non-screening mammography between age 50–59, in all included women. [file 12889_2023_16589_MOESM1_ESM.pdf]

Supplementary material to: German mammography screening program: adherence, characteristics of (non-)participants and utilization of non-screening mammography - a longitudinal analysis

Miriam Heinig[1], Wiebke Schäfer[1], Ingo Langner[1], Hajo Zeeb[2,3], Ulrike Haug[1,3]
1 Department of Clinical Epidemiology, Leibniz Institute for Prevention Research and Epidemiology – BIPS, Germany
2 Department of Prevention and Evaluation, Leibniz Institute for Prevention Research and Epidemiology – BIPS, Bremen, Germany
3 Faculty of Human and Health Sciences, University of Bremen, Germany

Figure A1 Flowchart of selection of the study population
Table A1 Distribution of women with birth year 1959 and at least one day of insurance in 2009 excluded due to lack of continuous insurance from 2007-2018 according to educational level
Table A2 Utilization of other preventive measures and prevalence of comorbidities and other characteristics among women excluded due to lack of continuous insurance from 2007-2018 with information on covariates at age 48 and/or 49
Table A3 Prevalence of comorbidities and other characteristics, stratified by ten-year adherence to mammography screening
Tables A4-A5 Utilization of other preventive measures and prevalence of comorbidities and other characteristics at age 55 and 59, stratified by ten-year adherence to mammography screening
Tables A6 Utilization of other preventive measures and prevalence of comorbidities and other characteristics, stratified by ten-year adherence to mammography screening (one-time only vs. 1–2 times)
Tables A7 Supplemental analysis regarding educational level and adherence to mammography screening
Tables A8 Characterization of all included women by age and educational level
Tables A9 Utilization of other preventive measures and prevalence of comorbidities and other characteristics in all included women
Tables A10 Total number of non-screening mammographies, and characterization of the use of non-screening mammographies in women with at least one non-screening mammography between age 50–59, in all included women

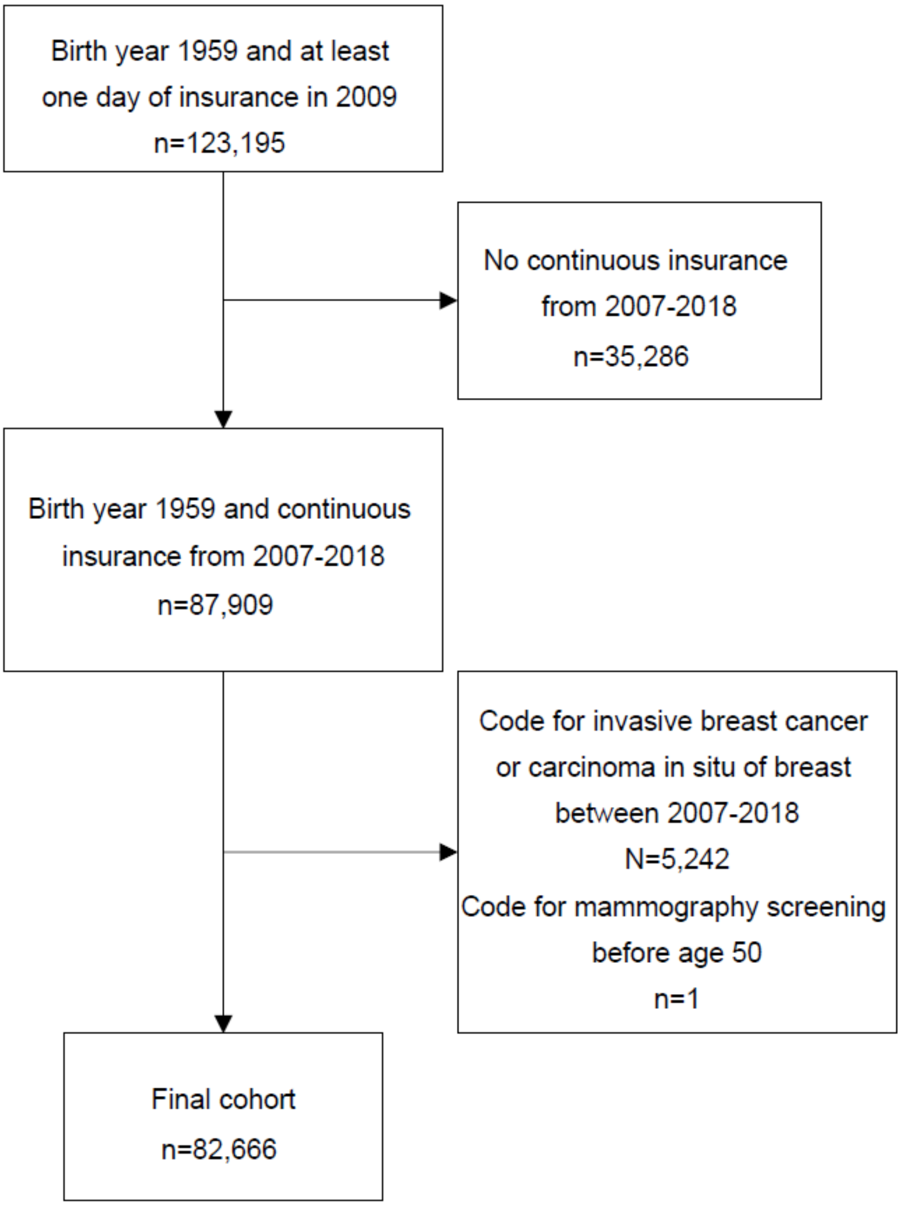

Figure A1 Flowchart of selection of the study population

Table A1 Distribution of women with birth year 1959 and at least one day of insurance in 2009 excluded due to lack of continuous insurance from 2007-2018 according to educational level

	Excluded		
	**N**	**%**	**95% CI**
	**35,286**	**(100%)**	
**Education**			
Basic secondary degree/secondary degree or missing information	26,301	(74.5%)	(74.1, 75.0)
Higher education	8,985	(25.5%)	(25.0, 25.9)

CI = confidence interval

Table A2 Utilization of other preventive measures and prevalence of comorbidities and other characteristics among women excluded due to lack of continuous insurance from 2007-2018 with information on covariates at age 48 and/or 49

	Excluded (with covariate information)		
	**N**	**%**	**95% CI**
	**26,998**	**(100%)**	
**Other screening and preventive measures[a]**			
Cervical cancer screening (PAP test)[b]	18,220	(67.5%)	(66.9, 68.0)
Skin cancer screening[c]	1,807	(6.7%)	(6.4, 7.0)
Health checkup	10,241	(37.9%)	(37.4, 38.5)
Influenza vaccination	4,579	(17.0%)	(16.5, 17.4)
Any preventive measure	20,853	(77.2%)	(76.7, 77.7)
**Comorbidities**			
Any severe comorbidity[d]	2,047	(7.6%)	(7.3, 7.9)
Any treatment for coronary heart disease	537	(2.0%)	(1.8, 2.2)
Treatment for hypertension	3,609	(13.4%)	(13.0, 13.8)
Obesity	3,083	(11.4%)	(11.0, 11.8)
Glaucoma	1,129	(4.2%)	(3.9, 4.4)
Treatment with antidepressants	1,021	(3.8%)	(3.6, 4.0)
Treatment with antipsychotics	221	(0.8%)	(0.7, 0.9)
**Other characteristics**			
Alcohol abuse	571	(2.1%)	(2.0, 2.3)
Tobacco abuse	1,683	(6.2%)	(6.0, 6.5)
Menopausal hormone therapy	4,064	(15.1%)	(14.6, 15.5)
Family history of breast cancer	870	(3.2%)	(3.0, 3.4)
Use of non-screening mammography	4,886	(18.1%)	(17.6, 18.6)

CI = confidence interval, PAP = papanicolaou test

[a] Screening for colorectal cancer with the fecal occult blood test is offered from age 50 (age 55: additional offer of screening colonoscopy) and therefore not included here.

[b] As part of screening for female cancers, cervical cancer screening can be combined with physical breast examination.

[c] Codes for skin cancer screening were available starting in July 2008 only.

[d] Any of the following: Liver disease, coronary heart disease, congestive heart failure, myocardial infarction, stroke, COPD, Hepatitis (B, C), renal insufficiency (terminal), diabetes with organ damage, treated HIV, dementia, plegia.

Table A2 (cont.)

	Excluded (with covariate information)		
	**N**	**%**	**95% CI**
	**26,998**	**(100%)**	
**Comorbidities**			
Coronary heart disease	196	(0.7%)	(0.6, 0.8)
Congestive heart failure	87	(0.3%)	(0.3, 0.4)
Myocardial infarction	22	(0.1%)	(0.1, 0.1)
Stroke	45	(0.2%)	(0.1, 0.2)
Any treatment for coronary heart disease	537	(2.0%)	(1.8, 2.2)
… with ASS	42	(0.2%)	(0.1, 0.2)
… only with ASS	105	(0.4%)	(0.3, 0.5)
… ASS + other	131	(0.5%)	(0.4, 0.6)
… with anticoagulants	110	(0.4%)	(0.3, 0.5)
… with statins	357	(1.3%)	(1.2, 1.5)
Asthma	1,332	(4.9%)	(4.7, 5.2)
Liver disease	913	(3.4%)	(3.2, 3.6)
COPD	545	(2.0%)	(1.9, 2.2)
Hepatitis (B, C)	17	(0.1%)	(0.0, 0.1)
Severe liver disease	46	(0.2%)	(0.1, 0.2)
Renal insufficiency, terminal	41	(0.2%)	(0.1, 0.2)
Diabetes, treated	489	(1.8%)	(1.7, 2.0)
Diabetes with organ complications	31	(0.1%)	(0.1, 0.2)
Treatment with immunosuppressive drugs	495	(1.8%)	(1.7, 2.0)
HIV, treated	7	(0%)	(0.0, 0.1)
Dementia	43	(0.2%)	(0.1, 0.2)
Paraplegia, hemiplegia	213	(0.8%)	(0.7, 0.9)

CI = confidence interval, ASS = acetylsalicylic acid, COPD = chronic obstructive pulmonary disease, HIV = human immunodeficiency virus

Table A3 Prevalence of comorbidities and other characteristics, stratified by ten-year adherence to mammography screening

	Number of participations in mammography screening											
	Never			1–2 times			3–4 times			5–6 times		
	**N**	**%**	**95% CI**	**N**	**%**	**95% CI**	**N**	**%**	**95% CI**	**N**	**%**	**95% CI**
	**22,786**	**(27.6%)**	**(27.3, 27.9)**	**12,521**	**(15.1%)**	**(14.9, 15.4)**	**26,166**	**(31.7%)**	**(31.3, 32.0)**	**21,193**	**(25.6%)**	**(25.3, 25.9)**
**Comorbidities**												
Asthma	1,087	(4.8%)	(4.5, 5.1)	723	(5.8%)	(5.4, 6.2)	1,633	(6.2%)	(6.0, 6.5)	1,235	(5.8%)	(5.5, 6.2)
Any treatment for coronary heart disease	427	(1.9%)	(1.7, 2.1)	245	(2.0%)	(1.7, 2.2)	559	(2.1%)	(2.0, 2.3)	458	(2.2%)	(2.0, 2.4)
… with ASS	43	(0.2%)	(0.1, 0.3)	17	(0.1%)	(0.1, 0.2)	50	(0.2%)	(0.1, 0.3)	38	(0.2%)	(0.1, 0.2)
… only with ASS	84	(0.4%)	(0.3, 0.5)	53	(0.4%)	(0.3, 0.6)	95	(0.4%)	(0.3, 0.4)	81	(0.4%)	(0.3, 0.5)
… ASS + other	113	(0.5%)	(0.4, 0.6)	67	(0.5%)	(0.4, 0.7)	129	(0.5%)	(0.4, 0.6)	107	(0.5%)	(0.4, 0.6)
… with anticoagulants	94	(0.4%)	(0.3, 0.5)	42	(0.3%)	(0.2, 0.5)	112	(0.4%)	(0.4, 0.5)	67	(0.3%)	(0.2, 0.4)
… with statins	274	(1.2%)	(1.1, 1.4)	167	(1.3%)	(1.1, 1.6)	370	(1.4%)	(1.3, 1.6)	342	(1.6%)	(1.5, 1.8)
Congestive heart failure	50	(0.2%)	(0.2, 0.3)	31	(0.2%)	(0.2, 0.4)	48	(0.2%)	(0.1, 0.2)	42	(0.2%)	(0.1, 0.3)
COPD	407	(1.8%)	(1.6, 2.0)	264	(2.1%)	(1.9, 2.4)	497	(1.9%)	(1.7, 2.1)	391	(1.8%)	(1.7, 2.0)
Coronary heart disease	162	(0.7%)	(0.6, 0.8)	105	(0.8%)	(0.7, 1.0)	191	(0.7%)	(0.6, 0.8)	147	(0.7%)	(0.6, 0.8)
Dementia	26	(0.1%)	(0.1, 0.2)	14	(0.1%)	(0.1, 0.2)	27	(0.1%)	(0.1, 0.2)	15	(0.1%)	(0.0, 0.1)
Diabetes, treated	395	(1.7%)	(1.6, 1.9)	230	(1.8%)	(1.6, 2.1)	464	(1.8%)	(1.6, 1.9)	327	(1.5%)	(1.4, 1.7)
Diabetes with organ complications	22	(0.1%)	(0.1, 0.1)	9	(0.1%)	(0.0, 0.1)	19	(0.1%)	(0.0, 0.1)	14	(0.1%)	(0.0, 0.1)
Hepatitis (B, C)	14	(0.1%)	(0.0, 0.1)	6	(0%)	(0.0, 0.1)	14	(0.1%)	(0.0, 0.1)	10	(0%)	(0.0, 0.1)
HIV, treated	9	(0%)	(0.0, 0.1)	5	(0%)	(0.0, 0.1)	4	(0%)	(0.0, 0)	6	(0%)	(0.0, 0.1)
Liver disease	655	(2.9%)	(2.7, 3.1)	445	(3.6%)	(3.2, 3.9)	846	(3.2%)	(3.0, 3.5)	732	(3.5%)	(3.2, 3.7)
Myocardial infarction	15	(0.1%)	(0.0, 0.1)	8	(0.1%)	(0.0, 0.1)	19	(0.1%)	(0.0, 0.1)	19	(0.1%)	(0.1, 0.1)
Paraplegia, hemiplegia	209	(0.9%)	(0.8, 1.0)	107	(0.9%)	(0.7, 1.0)	171	(0.7%)	(0.6, 0.8)	109	(0.5%)	(0.4, 0.6)
Renal insufficiency, terminal	22	(0.1%)	(0.1, 0.1)	13	(0.1%)	(0.1, 0.2)	21	(0.1%)	(0.1, 0.1)	10	(0%)	(0.0, 0.1)
Severe liver disease	24	(0.1%)	(0.1, 0.2)	23	(0.2%)	(0.1, 0.3)	30	(0.1%)	(0.1, 0.2)	16	(0.1%)	(0.0, 0.1)
Stroke	42	(0.2%)	(0.1, 0.2)	18	(0.1%)	(0.1, 0.2)	34	(0.1%)	(0.1, 0.2)	33	(0.2%)	(0.1, 0.2)
Treatment with immunosuppressive drugs	298	(1.3%)	(1.2, 1.5)	185	(1.5%)	(1.3, 1.7)	449	(1.7%)	(1.6, 1.9)	345	(1.6%)	(1.5, 1.8)

**Other characteristics**												
Drug abuse	224	(1.0%)	(0.9, 1.1)	104	(0.8%)	(0.7, 1.0)	129	(0.5%)	(0.4, 0.6)	76	(0.4%)	(0.3, 0.4)
Use of non-screening mammography	3,717	(16.3%)	(15.8, 16.8)	2,270	(18.1%)	(17.5, 18.8)	5,441	(20.8%)	(20.3, 21.3)	3,910	(18.4%)	(17.9, 19.0)

CI = confidence interval, ASS = acetylsalicylic acid, COPD = chronic obstructive pulmonary disease, HIV = human immunodeficiency virus

Table A4 Utilization of other preventive measures and prevalence of comorbidities and other characteristics (age 53 and 54), stratified by ten-year adherence to mammography screening

	Number of participations in mammography screening											
	Never			1–2 times			3–4 times			5–6 times		
	N	%	95% CI	N	%	95% CI	N	%	95% CI	N	%	95% CI
	**22,786**	**(27.6%)**	**(27.3, 27.9)**	**12,521**	**(15.1%)**	**(14.9, 15.4)**	**26,166**	**(31.7%)**	**(31.3, 32.0)**	**21,193**	**(25.6%)**	**(25.3, 25.9)**
**Other screening and preventive measures[a]**												
Cervical cancer screening (PAP test)[b]	14,730	(64.6%)	(45.2, 46.5)	7,882	(63.0%)	(62.1, 63.8)	19,712	(75.3%)	(74.8, 75.9)	17,147	(80.9%)	(80.4, 81.4)
Skin cancer screening[c]	5,527	(24.3%)	(23.7, 24.8)	3,904	(31.2%)	(30.4, 32.0)	9,357	(35.8%)	(35.2, 36.3)	8,260	(39.0%)	(38.3, 39.6)
Health checkup	8,717	(38.3%)	(37.6, 38.9)	5,761	(46.0%)	(45.1, 46.9)	13,330	(50.9%)	(50.3, 51.5)	11,509	(54.3%)	(53.6, 55)
Influenza vaccination	2,201	(9.7%)	(9.3, 10.0)	1,527	(12.2%)	(11.6, 12.8)	4,519	(17.3%)	(16.8, 17.7)	4,213	(19.9%)	(19.3, 20.4)
FOBT	6,698	(29.4%)	(28.8, 30.0)	5,198	(41.5%)	(40.7, 42.4)	13,311	(50.9%)	(50.3, 51.5)	11,737	(55.4%)	(54.7, 56)
Any of these preventive measures	14,730	(64.6%)	(64.0, 65.3)	9,969	(79.6%)	(78.9, 80.3)	22,979	(87.8%)	(87.4, 88.2)	19,290	(91.0%)	(90.6, 91.4)
**Comorbidities**												
Any severe comorbidity[d]	2,451	(10.8%)	(10.4, 11.2)	1,463	(11.7%)	(11.1, 12.3)	2,958	(11.3%)	(10.9, 11.7)	2,356	(11.1%)	(10.7, 11.5)
Treatment for hypertension	4,442	(19.5%)	(19.0, 20.0)	2,730	(21.8%)	(21.1, 22.5)	6,584	(25.2%)	(24.6, 25.7)	5,505	(26.0%)	(25.4, 26.6)
Obesity	2,439	(10.7%)	(10.3, 11.1)	1,661	(13.3%)	(12.7, 13.9)	3,778	(14.4%)	(14.0, 14.9)	3,221	(15.2%)	(14.7, 15.7)
Glaucoma	890	(3.9%)	(3.7, 4.2)	606	(4.8%)	(4.5, 5.2)	1,511	(5.8%)	(5.5, 6.1)	1,418	(6.7%)	(6.4, 7.0)
Treatment with antidepressants	1,078	(4.7%)	(4.5, 5.0)	808	(6.5%)	(6.0, 6.9)	1,533	(5.9%)	(5.6, 6.1)	1,137	(5.4%)	(5.1, 5.7)
Treatment with antipsychotics	342	(1.5%)	(1.4, 1.7)	189	(1.5%)	(1.3, 1.7)	282	(1.1%)	(1.0, 1.2)	137	(0.6%)	(0.5, 0.8)
**Other characteristics**												
Alcohol abuse	551	(2.4%)	(2.2, 2.6)	319	(2.5%)	(2.3, 2.8)	388	(1.5%)	(1.3, 1.6)	233	(1.1%)	(1.0, 1.2)
Tobacco abuse	1,639	(7.2%)	(6.9, 7.5)	1,064	(8.5%)	(8.0, 9.0)	1,933	(7.4%)	(7.1, 7.7)	1,464	(6.9%)	(6.6, 7.3)
Menopausal hormone therapy	3,290	(14.4%)	(14.0, 14.9)	2,584	(20.6%)	(19.9, 21.4)	6,216	(23.8%)	(23.2, 24.3)	5,378	(25.4%)	(24.8, 26.0)
Family history of breast cancer	1,711	(7.5%)	(7.2, 7.9)	833	(6.7%)	(6.2, 7.1)	1,483	(5.7%)	(5.4, 6.0)	921	(4.3%)	(4.1, 4.6)
Use of non-screening mammography	3,496	(15.3%)	(14.9, 15.8)	1,542	(12.3%)	(11.8, 12.9)	897	(3.4%)	(3.2, 3.7)	183	(0.9%)	(0.7, 1.0)

CI = confidence interval, PAP = papanicolaou test, FOBT = fecal occult blood test

[a] Screening for colorectal cancer with the fecal occult blood test is offered from age 50 (age 55: additional offer of screening colonoscopy).

[b] As part of screening for female cancers, cervical cancer screening can be combined with physical breast examination.

[c] Codes for skin cancer screening were available starting in July 2008 only.

[d] Any of the following: Liver disease, coronary heart disease, congestive heart failure, myocardial infarction, stroke, COPD, Hepatitis (B, C), renal insufficiency (terminal), diabetes with organ damage, treated HIV, dementia, plegia.

Table A5 Utilization of other preventive measures and prevalence of comorbidities and other characteristics (age 57 and 58), stratified by ten-year adherence to mammography screening

	Number of participations in mammography screening											
	Never			1–2 times			3–4 times			5–6 times		
	N	%	95% CI	N	%	95% CI	N	%	95% CI	N	%	95% CI
	**22,786**	**(27.6%)**	**(27.3, 27.9)**	**12,521**	**(15.1%)**	**(14.9, 15.4)**	**26,166**	**(31.7%)**	**(31.3, 32.0)**	**21,193**	**(25.6%)**	**(25.3, 25.9)**
**Other screening and preventive measures**												
Cervical cancer screening (PAP test)[a]	8,943	(39.2%)	(38.6, 39.9)	6,847	(54.7%)	(53.8, 55.6)	17,682	(67.6%)	(67, 68.1)	16,046	(75.7%)	(75.1, 76.3)
Skin cancer screening[b]	5,746	(25.2%)	(24.7, 25.8)	3,846	(30.7%)	(29.9, 31.5)	9,580	(36.6%)	(36, 37.2)	8,766	(41.4%)	(40.7, 42.0)
Health checkup	9,030	(39.6%)	(39, 40.3)	5,941	(47.4%)	(46.6, 48.3)	13,370	(51.1%)	(50.5, 51.7)	11,828	(55.8%)	(55.1, 56.5)
Influenza vaccination	1,580	(6.9%)	(6.6, 7.3)	1,036	(8.3%)	(7.8, 8.8)	3,036	(11.6%)	(11.2, 12.0)	3,045	(14.4%)	(13.9, 14.8)
FOBT	3,944	(17.3%)	(16.8, 17.8)	2,967	(23.7%)	(23.0, 24.4)	7,300	(27.9%)	(27.4, 28.4)	6,613	(31.2%)	(30.6, 31.8)
Screening colonoscopy	920	(4.0%)	(3.8, 4.3)	786	(6.3%)	(5.9, 6.7)	2,221	(8.5%)	(8.2, 8.8)	2,198	(10.4%)	(10.0, 10.8)
Any of these preventive measures	14,080	(61.8%)	(61.2, 62.4)	9,479	(75.7%)	(74.9, 76.4)	22,049	(84.3%)	(83.8, 84.7)	18,895	(89.2%)	(88.7, 89.6)
**Comorbidities**												
Any severe comorbidity[c]	3,309	(14.5%)	(14.1, 15.0)	2,096	(16.7%)	(16.1, 17.4)	4,174	(16.0%)	(15.5, 16.4)	3,315	(15.6%)	(15.2, 16.1)
Treatment for hypertension	5,810	(25.5%)	(24.9, 26.1)	3,549	(28.3%)	(27.6, 29.1)	8,384	(32.0%)	(31.5, 32.6)	7,020	(33.1%)	(32.5, 33.8)
Obesity	2,922	(12.8%)	(12.4, 13.3)	1,987	(15.9%)	(15.2, 16.5)	4,566	(17.5%)	(17.0, 17.9)	4,024	(19.0%)	(18.5, 19.5)
Glaucoma	1,015	(4.5%)	(4.2, 4.7)	678	(5.4%)	(5.0, 5.8)	1,676	(6.4%)	(6.1, 6.7)	1,625	(7.7%)	(7.3, 8.0)
Treatment with antidepressants	1,229	(5.4%)	(5.1, 5.7)	870	(6.9%)	(6.5, 7.4)	1,747	(6.7%)	(6.4, 7.0)	1,205	(5.7%)	(5.4, 6.0)
Treatment with antipsychotics	378	(1.7%)	(1.5, 1.8)	213	(1.7%)	(1.5, 1.9)	314	(1.2%)	(1.1, 1.3)	172	(0.8%)	(0.7, 0.9)
**Other characteristics**												
Alcohol abuse	627	(2.8%)	(2.5, 3.0)	345	(2.8%)	(2.5, 3.1)	493	(1.9%)	(1.7, 2.1)	284	(1.3%)	(1.2, 1.5)
Tobacco abuse	2,025	(8.9%)	(8.5, 9.3)	1,303	(10.4%)	(9.9, 11.0)	2,383	(9.1%)	(8.8, 9.5)	1,723	(8.1%)	(7.8, 8.5)
Menopausal hormone therapy	3,290	(14.4%)	(14.0, 14.9)	2,548	(20.3%)	(19.7, 21.1)	6,279	(24.0%)	(23.5, 24.5)	5,482	(25.9%)	(25.3, 26.5)
Family history of breast cancer	2,054	(9.0%)	(8.6, 9.4)	1,039	(8.3%)	(7.8, 8.8)	1,844	(7.0%)	(6.7, 7.4)	1,161	(5.5%)	(5.2, 5.8)
Use of non-screening mammography	2,936	(12.9%)	(12.5, 13.3)	1,185	(9.5%)	(9.0, 10.0)	758	(2.9%)	(2.7, 3.1)	127	(0.6%)	(0.5, 0.7)

CI = confidence interval, PAP = papanicolaou test, FOBT = fecal occult blood test

[a] As part of screening for female cancers, cervical cancer screening can be combined with physical breast examination.

[b] Codes for skin cancer screening were available starting in July 2008 only.

[c] Any of the following: Liver disease, coronary heart disease, congestive heart failure, myocardial infarction, stroke, COPD, Hepatitis (B, C), renal insufficiency (terminal), diabetes with organ damage, treated HIV, dementia, plegia.

Table A6 Utilization of other preventive measures and prevalence of comorbidities and other characteristics, stratified by ten-year adherence to mammography screening (one-time only vs. 1–2 times)

	Number of participations in mammography screening					
	One-time only			1–2 times		
	N	%	95% CI	N	%	95% CI
	**6,402**	**(7.7%)**	**(7.6, 7.9)**	**12,521**	**(15.1%)**	**(14.9, 15.4)**
**Other screening and preventive measures[a]**						
Cervical cancer screening (PAP test)[b]	4,445	(69.4%)	(68.3, 70.5)	8,946	(71.4%)	(70.7, 72.7)
Skin cancer screening[c]	436	(6.8%)	(6.2, 7.5)	840	(6.7%)	(6.3, 7.2)
Health checkup	2,527	(39.5%)	(38.3, 40.7)	5,056	(40.4%)	(39.5, 41.2)
Influenza vaccination	873	(13.6%)	(12.8, 14.5)	1,819	(14.5%)	(13.9, 15.2)
Any of these preventive measures	5,133	(80.2%)	(79.2, 81.1)	10,226	(81.7%)	(81.0, 82.3)
**Comorbidities**						
Any severe comorbidity[d]	508	(7.9%)	(7.3, 8.6)	970	(7.7%)	(7.3, 8.2)
Treatment for hypertension	682	(10.7%)	(11.3, 12.8)	1,570	(12.5%)	(12.0, 13.1)
Obesity	744	(11.6%)	(10.9, 12.4)	1,468	(11.7%)	(11.2, 12.3)
Glaucoma	263	(4.1%)	(3.6, 4.6)	512	(4.1%)	(3.8, 4.5)
Treatment with antidepressants	292	(4.6%)	(4.1, 5.1)	598	(4.8%)	(4.4, 5.2)
Treatment with antipsychotics	69	(1.1%)	(0.9, 1.4)	142	(1.1%)	(1.0, 1.3)
**Other characteristics**						
Alcohol abuse	137	(2.1%)	(1.8, 2.5)	271	(2.2%)	(1.9, 2.4)
Tobacco abuse	380	(5.9%)	(5.4, 6.5)	771	(6.2%)	(5.7, 6.6)
Menopausal hormone therapy	916	(14.3%)	(13.5, 15.2)	1,925	(15.4%)	(14.8, 16.0)
Family history of breast cancer	227	(3.5%)	(3.1, 4.0)	434	(3.5%)	(3.2, 3.8)
Use of diagnostic mammography	1,098	(17.2%)	(16.2, 18.1)	2,270	(18.1%)	(1.9, 2.4)

CI = confidence interval, PAP = papanicolaou test

[a] Screening for colorectal cancer with the fecal occult blood test is offered from age 50 (age 55: additional offer of screening colonoscopy) and therefore not included here.

[b] As part of screening for female cancers, cervical cancer screening can be combined with physical breast examination.

[c] Codes for skin cancer screening were available starting in July 2008 only.

[d] Any of the following: Liver disease, coronary heart disease, congestive heart failure, myocardial infarction, stroke, COPD, Hepatitis (B, C), renal insufficiency (terminal), diabetes with organ damage, treated HIV, dementia, plegia.

A7 Supplemental analysis regarding educational level and mammography screening participation among excluded women

In our main analysis, women without continuous insurance from 2007–2018 were excluded. The proportion of women with higher education was about 10 percentage points lower among excluded women compared to included women. We therefore conducted a supplemental analysis in order to assess whether the exclusion of women without continuous insurance affected our estimation of participation and non-participation in the mammography screening program. In this analysis, we compared all women with continuous insurance until at least 2013 (Table S3A, n=91,759, all other inclusion criteria as shown in Figure S1 were also applied but restricted to 2007–2013 where applicable) and women who were excluded in the main analysis due to lack of continuous insurance with continuous insurance until at least 2013 (i.e., a subset of all women with continuous insurance until 2013, Table S3B, n=7,616). We categorized the women according to their participation in mammography screening as described in the main analysis, except from 2009 to 2013 (Never, 1–2 times, 3 times.) Among all women with continuous insurance until at least 2013 (Table S3A), and women excluded in the main analysis due to lack of continuous insurance until 2018 (Table S3B), there are no differences in the overall distribution according to adherence group. Furthermore, in both analyses the proportion of women with higher education was similar across all adherence groups.

Table A7A Distribution of women with continuous insurance from 2007 until at least 2013 according to five-year adherence to mammography screening and characterization of adherence groups by educational level

	**Number of participations in mammography screening**								
	**Never**			**1-2 times**			**3 times**		
	**N**	**%**	**95% CI**	**N**	**%**	**95% CI**	**N**	**%**	**95% CI**
	**29,699**	**(32.4%)**	**(32.1, 32.7)**	**50,646**	**(55.2%)**	**(54.9, 55.5)**	**11,414**	**(12.4%)**	**(12.2, 12.7)**
**Education [a]**									
Basic secondary degree/secondary degree or missing	19,266	(64.9%)	(64.3, 65.4)	32,487	(64.1%)	(63.7, 64.6)	7,306	(64.0%)	(63.1, 64.9)
Higher education	10,433	(35.1%)	(34.6, 35.7)	18,159	(35.9%)	(35.4, 36.3)	4,108	(36.0%)	(35.1, 36.9)

CI = confidence interval

Table A7B Distribution of women excluded due to lack of continuous insurance from 2007-2018 with continuous insurance from 2007 until at least 2013 according to five-year adherence to mammography screening and characterization of adherence groups by educational level

	Number of participations in mammography screening								
	**Never**			**1-2 times**			**3 times**		
	**N**	**%**	**95% CI**	**N**	**%**	**95% CI**	**N**	**%**	**95% CI**
	**2,573**	**(33.8%)**	**(32.7, 34.9)**	**4,208**	**(55.3%)**	**(54.1, 56.4)**	**835**	**(11.0%)**	**(10.3, 11.7)**
**Education [a]**									
Basic secondary degree/secondary degree or missing	1,887	(73.3%)	(71.6, 75.0)	3,123	(74.2%)	(72.9, 75.5)	632	(75.7%)	(72.7, 78.5)
Higher education	686	(26.7%)	(25.0, 28.4)	1,085	(25.8%)	(24.5, 27.1)	203	(24.3%)	(21.5, 27.3)

CI = confidence interval

Table A8 Characterization of all included women by age and educational level

	All women		
	**N**	**%**	**95% CI**
	**82,666**	**(100%)**	
**Age at first screen**			
Mean (SD)	51.5	(1.7)	(51.4, 51.5)
Median (IQR)	51.0	(50-52)	-
**Education**			
Basic secondary degree/secondary degree or missing information	52,515	(63.5%)	(63.2, 63.9)
Higher education	30,151	(36.5%)	(36.1, 36.8)

CI = confidence interval, SD = standard deviation, IQR = interquartile range, N/A = not applicable

Table A9 Utilization of other preventive measures and prevalence of comorbidities and other characteristics in all included women

	All women		
	**N**	**%**	**95% CI**
	**82,666**	**(100%)**	
**Other screening and preventive measures[a]**			
Cervical cancer screening (PAP test)[b]	60,253	(72.9%)	(72.6, 73.2)
Skin cancer screening[c]	5,938	(7.2%)	(7.0, 7.4)
Health checkup	34,150	(41.3%)	(41.0, 41.6)
Influenza vaccination	13,707	(16.6%)	(16.3, 16.8)
Any of these preventive measures	67,528	(81.7%)	(81.4, 81.9)
**Comorbidities**			
Any severe comorbidity[d]	5,942	(7.2%)	(7.0, 7.4)
Treatment for hypertension	11,187	(13.5%)	(13.3, 13.8)
Obesity	9,923	(12.0%)	(11.8, 12.2)
Glaucoma	3,928	(4.8%)	(4.6, 4.9)
Treatment with antidepressants	3,318	(4.0%)	(3.9, 4.1)
Treatment with antipsychotics	763	(0.9%)	(0.9, 1.0)
**Other characteristics**			
Alcohol abuse	1,201	(1.5%)	(1.4, 1.5)
Tobacco abuse	4,794	(5.8%)	(5.6, 6.0)
Menopausal hormone therapy	12,902	(15.6%)	(15.4, 15.9)
Family history of breast cancer	2,873	(3.5%)	(3.4, 3.6)
Use of non-screening mammography	15,338	(18.6%)	(18.3, 18.8)

CI = confidence interval, PAP = papanicolaou test

[a] Screening for colorectal cancer with the fecal occult blood test is offered from age 50 (age 55: additional offer of screening colonoscopy) and therefore not included here.
[b] As part of screening for female cancers, cervical cancer screening can be combined with physical breast examination.
[c] Codes for skin cancer screening were available starting in July 2008 only.
[d] Any of the following: Liver disease, coronary heart disease, congestive heart failure, myocardial infarction, stroke, COPD, Hepatitis (B, C), renal insufficiency terminal), diabetes with organ damage, treated HIV, dementia, plegia.

Table A10 Total number of non-screening mammographies, and characterization of the use of non-screening mammographies in women with at least one non-screening mammography between age 50–59, in all included women

	All women		
	N	%	95% CI
**Total number of non-screening mammographies between age 50–59**			
	82,666		
≥1 mammography	15,830	(19.1%)	(18.9, 19.4)
≥2 mammography	7,627	(9.2%)	(9.0, 9.4)
≥3 mammography	4,990	(6.0%)	(5.9, 6.2)
**Among those with ≥1 non-screening mammography**			
	15,830		
Distribution of the total number of non-screening mammographies			
mean (SD)	2.3	(1.9)	(2.3-2.3)
median (IQR)	1.0	(1.0-3.0)	-
1 mammography	8,203	(51.8%)	(51.0, 52.6)
2-3 mammographies	4,173	(26.4%)	(25.7, 27.1)
>3 mammographies	3,454	(21.8%)	(21.2, 22.5)
**Among those with ≥2 non-screening mammography**			
	7,627		
Time interval between non-screening mammographies			
mean (SD), years	2.0	(1.2)	(2.0-2.0)
median (IQR), years	1.9	(1.1-2.3)	-

CI = confidence interval, SD = standard deviation, IQR = interquartile range
